# Design and Biological Evaluation of Monoterpene-Conjugated *(S)*-2-Ethoxy-3-(4-(4-hydroxyphenethoxy)phenyl)propanoic Acids as New Dual PPARα/γ Agonists

**DOI:** 10.3390/molecules30244775

**Published:** 2025-12-14

**Authors:** Sergey A. Borisov, Mikhail E. Blokhin, Yulia V. Meshkova, Maria K. Marenina, Nataliya A. Zhukova, Sophia V. Pavlova, Anastasiya V. Lastovka, Vladislav V. Fomenko, Igor P. Zhurakovsky, Olga A. Luzina, Mikhail V. Khvostov, Dmitry A. Kudlay, Nariman F. Salakhutdinov

**Affiliations:** 1 N. N. Vorozhtsov Novosibirsk Institute of Organic Chemistry, Siberian Branch of the Russian Academy of Sciences, Akademika Lavrentieva Ave. 9, 630090 Novosibirsk, Russia; mblokhin@nioch.nsc.ru (M.E.B.); meshkova@nioch.nsc.ru (Y.V.M.); mareninamk@nioch.nsc.ru (M.K.M.); lastovka@nioch.nsc.ru (A.V.L.); fomenko@nioch.nsc.ru (V.V.F.); luzina@nioch.nsc.ru (O.A.L.); mihail.hvostov@gmail.com (M.V.K.); anvar@nioch.nsc.ru (N.F.S.); 2 Institute of Cytology and Genetics, Siberian Branch of Russian Academy of Sciences, Akademika Lavrentieva Ave. 10, 630090 Novosibirsk, Russia; spav@bionet.nsc.ru; 3 Department of Pathological Physiology and Clinical Pathophysiology, Faculty of Medicine, Novosibirsk State Medical University, Krasny Pr-t. 52, 630091 Novosibirsk, Russia; murash2003@yandex.ru; 4 V. Zelman Faculty for the Medicine and Psychology, Novosibirsk State University, Pirogova Str. 1, 630090 Novosibirsk, Russia; 5 Institute of Pharmacy, I. M. Sechenov First Moscow State Medical University, St. Trubetskaya 8/2, 119991 Moscow, Russia; d624254@gmail.com; 6 Faculty of Bioengineering and Bioinformatics, Lomonosov Moscow State University, Leninskie Gory 1/73, 119234 Moscow, Russia

**Keywords:** dual PPARα/γ agonists, metabolic syndrome, type 2 diabetes mellitus, hypoglycemic activity, hypolipidemic activity, monoterpenoids

## Abstract

Metabolic syndrome, a collective term for lipid and carbohydrate disorders in the organism, is the primary cause of type 2 diabetes mellitus development and its associated systemic side effects. The current approach for the medical treatment of this condition usually requires multiple medications, targeting multiple pathophysiological pathways. A promising drug class in that regard is the dual PPARα/γ agonists, which impact both lipid and carbohydrate metabolism, yet to this day the vast majority of them have not passed the clinical trials, due to potential toxicity risks. In the present study we synthesized and tested a series of monoterpene-substituted *(S)*-2-ethoxy-3-(4-(4-hydroxyphenethoxy)phenyl)propanoic acids as potentially effective and safe novel dual PPARα/γ agonists. In vitro studies showed that nearly all of the tested compounds were sufficiently active towards both PPARα and PPARγ. All compounds were tested in vivo, using C57BL/6 A^y^/_a_ mice with T2DM symptoms, in order to evaluate their impact on carbohydrate and lipid metabolism. The most promising of them was found to be compound **5h**, containing a cumin fragment, which showed pronounced hypoglycemic activity by boosting tissue insulin sensitivity and hypolipidemic effects manifested by reductions in fat tissue mass and blood triglyceride levels, while simultaneously displaying a relatively safe profile.

## 1. Introduction

Metabolic syndrome (MetS) represents a cluster of interrelated abnormalities, including obesity, hypertension, dyslipidemia, and elevated blood glucose, which collectively significantly elevate the risk of developing cardiovascular diseases, type 2 diabetes mellitus, and other conditions. The term “metabolic” specifically refers to the underlying disruptions in the body’s essential processes, such as lipid and carbohydrate metabolism [1].

A cornerstone of MetS management involves lifestyle modifications, which encompass weight reduction, increased physical activity, and a low-calorie diet. However, these measures frequently prove insufficient, creating a need for adjunct pharmacotherapy that can simultaneously target multiple pathophysiological pathways [1]. This often leads to polypharmacy—the concurrent use of several medications—which can adversely affect patient adherence and increase the xenobiotic burden on the body. Therefore, the development of pharmacological agents capable of simultaneously influencing multiple pathophysiological targets of the metabolic syndrome is of great importance. Such targets include peroxisome proliferator-activated receptors (PPAR). Over the past two decades, these receptors have been regarded as valuable pharmacological targets, the activation of which can normalize metabolic dysfunctions and mitigate cardiovascular risk factors associated with type 2 diabetes and hyperlipidemia. The structures of the PPAR subtypes (α, γ, β/δ) are generally similar and quite typical of the nuclear receptor superfamily. They share a DNA-binding domain (D), a phosphorylation domain (A/B) and a cofactor activation domain (C) while the ligand-binding domain (E/F) is the one that has the most differences between the subtypes and determines which substances will be able to bind to the receptor [2].

The most extensively studied PPAR subtypes are PPAR-α and PPAR-γ. PPAR-α agonists (known as fibrates) can ameliorate dyslipidemia while PPAR-γ agonists (glitazones) reduce insulin resistance, functioning as peripheral insulin sensitizers.

The development of dual PPAR ligands, which integrate the beneficial effects of both α- and γ-agonists into a single molecule, has emerged as a promising therapeutic strategy for MetS. This concept is supported by substantial evidence demonstrating that PPAR co-agonists, termed glitazars, not only reduce insulin resistance but also correct abnormalities in fatty acid and lipoprotein metabolism [3]. Notably, the effects of these dual agonists were more pronounced than those of mono-agonists targeting individual receptors [4]. In addition to their impact on lipid and glucose homeostasis, dual agonists exhibit anti-inflammatory and anti-proliferative properties in vascular tissues.

**Figure 1:**
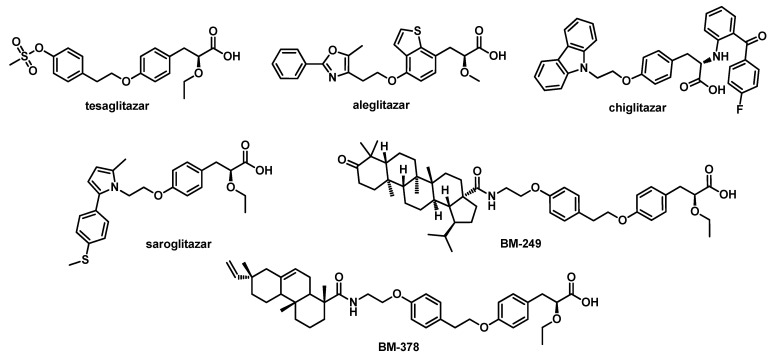
Structures of glitazars.

Based on the results of these studies, several pharmaceutical companies have developed and tested dual PPAR α/γ agonists, demonstrating promising results in animal studies. However, most of these compounds—such as tesaglitazar and other [5,6,7] (Figure 1)—failed to gain regulatory approval for clinical use due to safety concerns and a profile of adverse effects.

Subsequent research efforts have focused on structural optimization, particularly by modifying the lipophilic moiety of the glitazar molecule with synthetic fragments. This approach has yielded more successful therapeutic agents. For instance, pharmacological data on newer compounds—aleglitazar [8], saroglitazar [9], and chiglitazar [10]—attest to their improved efficacy and safety profiles, with saroglitazar and chiglitazar being approved for clinical use in India and China, respectively.

These outcomes reinforce the hypothesis that targeted chemical modification of the lipophilic segment can enhance pharmacokinetic and pharmacodynamic properties without compromising receptor binding affinity. Structural analysis of glitazars reveals that the most common pharmacophore is *(S)*-2-ethoxy-3-phenylpropanoic acid, a fragment whose derivatives show optimal selectivity and safety in binding to PPAR-α and PPAR-γ receptors [11]. Given that PPARs are nuclear receptors highly expressed in the liver and adipose tissue, the lipophilic fragment is typically an aromatic or heterocyclic system connected to the pharmacophore core via a short, flexible linker. This linker is crucial as it provides the conformational flexibility needed for optimal interaction with amino acid residues in the receptor’s binding pocket [12]. An appropriate combination of the glitazar’s pharmacophoric core with a lipophilic moiety may yield a new generation of effective and safe dual PPAR agonists.

Natural compounds are well-established as platforms for the synthesis of medicinal drugs derived from them [13]. Experimental studies have demonstrated that plant-based drugs containing complex mixtures of natural terpenes can, upon oral administration, effectively inhibit the rise in atherogenic lipoproteins, total cholesterol, low-density lipoprotein (LDL), and triglycerides. Our previous research (Figure 1) has shown that the incorporation of triterpenic and diterpenic acid fragments as the variable moiety of *(S)*-2-ethoxy-3-(4-(4-hydroxyphenethoxy)phenyl)propanoic acid yields compounds BM-249 and BM-378 exhibiting moderate hypoglycemic and hypolipidemic properties [14,15].

The critical influence of the variable moiety’s structure on glitazar activity is further underscored by the observation that both extending the linker length and increasing the volume of the lipophilic group lead to diminished hypoglycemic and hypolipidemic effects [16]. This decline in binding affinity and efficacy is presumably due to steric hindrance and an unfavorable conformation of the molecule within the binding pocket of the PPAR receptor. In this context, the use of monoterpenoids as the variable component of a glitazar molecule presents a highly promising research direction.

Monoterpenoids constitute the largest group of plant secondary metabolites and include acyclic, monocyclic, and bicyclic structures. Their diverse chemical structures and biological activities make them a rich foundation for developing new drugs to treat a range of significant diseases [17].

In the context of metabolic syndrome, agents based on monoterpenoids are capable of reducing arterial blood pressure, blood glucose, and triglyceride levels [18,19].

Specific examples include α-limonene, perillyl alcohol, and geraniol, which have shown antidiabetic effects in streptozotocin-induced diabetic rats [20,21,22]. Furthermore, other derivatives, such as those based on myrcene and pinane scaffolds, have also been reported to positively influence blood glucose levels and lipid profiles [23,24].

**Figure 2:**
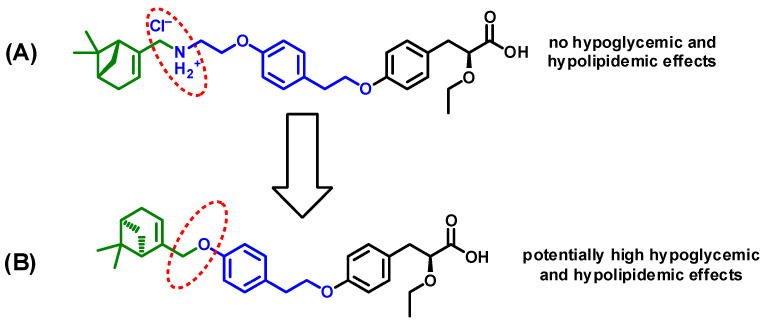
Structural evolution from an inactive to a proposed active glitazar. (**A**) The structure of a previously synthesized monoterpenoid amine derivative; (**B**) The proposed monoterpene-conjugated glitazar structure featuring direct conjugation via an ether bond.

In our previous study, replacing the tri- and diterpenoid moiety in the structure of *(S)*-3-(4-(4-(2-aminoethoxy)phenethoxy)phenyl)-2-ethoxypropanoic acid with a monoterpenoid motif was shown to result in a loss of hypoglycemic and hypolipidemic activity [14]. This effect is presumably due to the presence of a basic aliphatic amino group. At physiological pH, this group is protonated, which can reduce lipophilicity and disrupt optimal binding to PPARs (Figure 2).

Furthermore, the structures of glitazars illustrated in Figure 1 predominantly feature ether linkages or connections via nitrogen-containing heterocycles. For this reason, in the present study, we propose a novel design for glitazars incorporating monoterpenoids, wherein the natural and pharmacophoric fragments are directly conjugated without the mediation of an amino alcohol linker.

## 2. Results

### 2.1. Chemistry

**Scheme 1:**
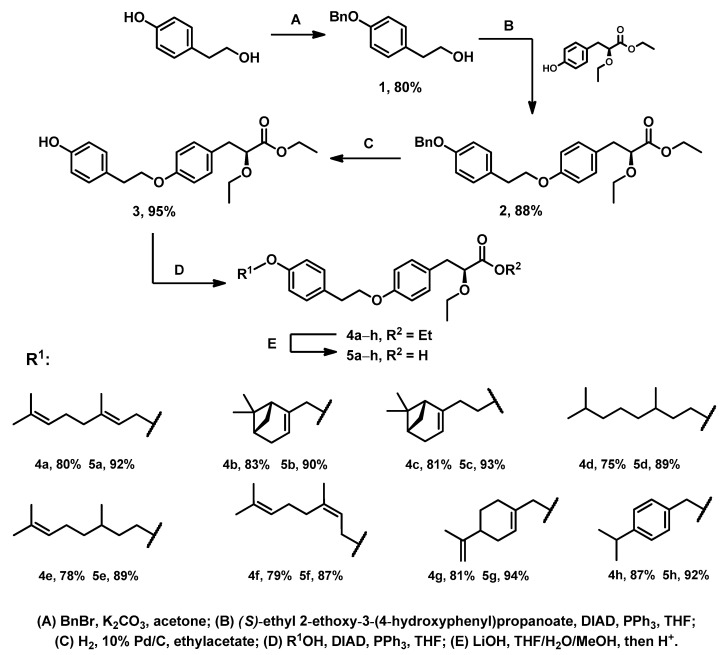
Synthesis of the key fragment **3**.

Monoterpene derivatives **5a**–**h** were synthesized in five steps from 4-(2-hydroxyethyl)phenol; the general scheme of the synthesis is shown in Scheme 1. Ethyl *(S)*-2-ethoxy-3-(4-hydroxyphenyl)propionate was synthesized according to the procedure described in [25]. In the first step, the phenolic hydroxyl group of 4-(2-hydroxyethyl)phenol was protected using a slight excess of benzyl bromide in acetone, with potassium carbonate as a base. The resulting benzyl ether **1** was reacted with ethyl *(S)*-2-ethoxy-3-(4-hydroxyphenyl)propionate under Mitsunobu conditions in THF, using PPh_3_ and DIAD in an inert atmosphere. The resulting ester **2** was purified by column chromatography. Debenzylation of the benzyl ether was carried out via hydrogenation in the presence of 10% palladium on carbon, following the method described in [16]. Phenol **3** was used in the next step without further purification.

The reaction of the corresponding monoterpene alcohol with phenol **3** was carried out under Mitsunobu conditions in the presence of DIAD and PPh_3_ in THF under an inert atmosphere. A 5% molar excess of phenol relative to the alcohol was used, which facilitated complete conversion of the alcohol and increased the efficiency of purification of the reaction mixture by column chromatography. Esters **4a**–**h** were isolated in high yields of 75–87%. After hydrolysis of the ester group with lithium hydroxide, a series of acids **5a**–**h** were obtained, also in high yields of 87–94%.

This versatile strategy enables the construction of potential glitazars through modifications of both the terpenoid acid and the linker attached to the pharmacophoric core. To meet the standards for biological assays, all final compounds were purified via column chromatography. Their purity, confirmed by HPLC and NMR spectroscopy, was determined to be at least 95%.

### 2.2. In Vitro Biological Testing

#### 2.2.1. MTT Assay

To determine the safe dose of the investigated compounds for in vitro assessment of their ability to act as agonists of PPARα and PPARγ receptors, an MTT assay was conducted. The results (Table 1) indicated that at the concentration of 100 μM, all compounds exhibited cytotoxicity, with cell viability below 10%. At 10 μM, the majority of compounds (except for **5a**, e.g., were also cytotoxic. At 1 μM, cell viability across all groups exceeded 95%, indicating the absence of cytotoxicity for the compounds at this concentration.

#### 2.2.2. PPARα and PPARγ Receptor Agonist Activity Evaluation

**Figure 3:**
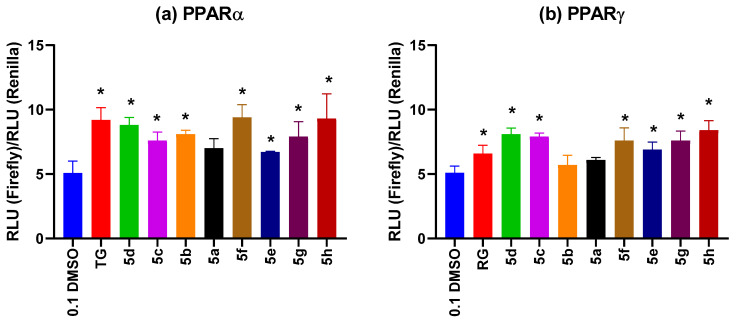
Luciferase assay of compounds **5a**–**h**: with tesaglitazar (TG) in CHO-PPARAα (**a**) and with rosiglitazone (RG) in CHO-PPARγ (**b**) (all compounds were in 1 µM); * Indicates a statistically significant difference (*p* ≤ 0.05) compared to control (0.1% DMSO). Statistical comparisons were performed using the Wilcoxon test with Bonferroni correction for multiple comparisons.

We tested the effect of the experimental compounds **5a**–**h** at 1 µM concentration (MTT-assay) on PPAR using reporter constructs expressing firefly luciferase under 3xPPRE DNA binding elements on transgenic cell lines CHO-hPPARα and CHO-hPPARγ [15] (Figure 3). Compound **5b** increases the activity of the luciferase reporter construct only in CHO-hPPARα cells and can be classified as a PPARa agonist. The activity of the reporter gene in the presence of compound **5a** was not significantly different from that of the control (0.1% DMSO) in any of the transgenic lines. The remaining studied substances can be categorized as dual agonists, as they increase the activity of the luciferase reporter construct in both transgenic cell lines.

### 2.3. In Vivo Experiments

#### 2.3.1. Body Mass Dynamics

**Figure 4:**
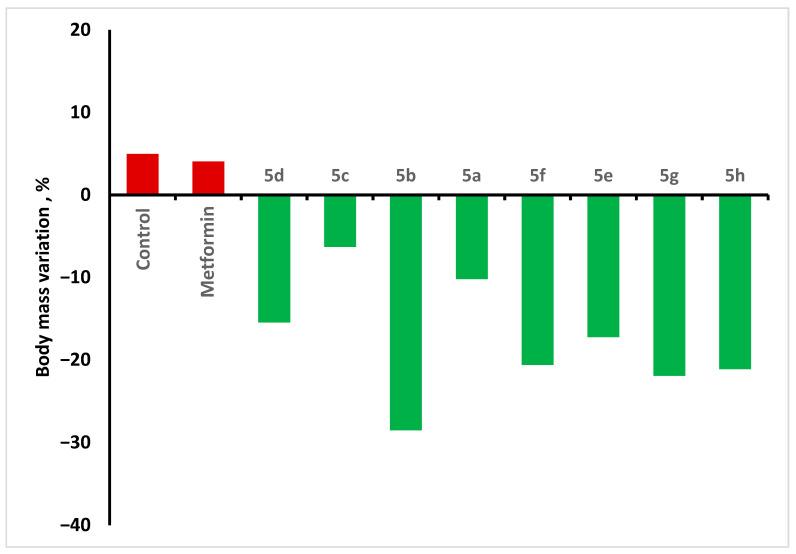
Dynamics of animal body mass. Data are presented as the percentage change in body mass at the end of the experiment relative to the initial mass. Group size n = 7.

In all conducted experiments, body weight was measured in animals once weekly throughout the entire duration of the investigated compounds’ administration. Based on the obtained data, indicators of body mass change in mice at the end of the experiment relative to the initial value were calculated. As evident from the presented diagram (Figure 4), the body weight of animals in the control group and in the group receiving the reference drug metformin exhibited a slight increase by the end of the experiment, while in mice treated with the investigated compounds it, conversely, decreased. The most substantial reduction in this parameter was observed in groups following administration of **5b**,**f**–**h**.

#### 2.3.2. OGTT

**Figure 5:**
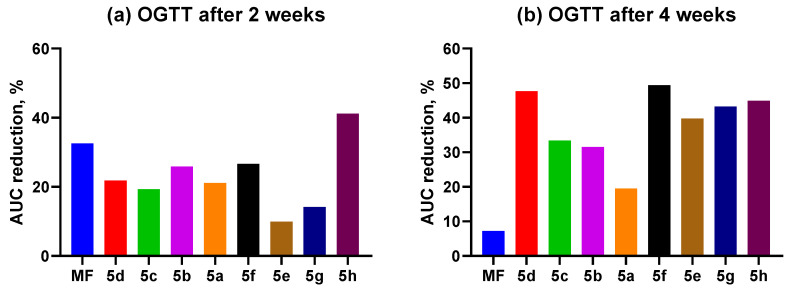
Results of OGTT conducted after 2 weeks (**a**) and 4 weeks (**b**) of compound administration. Data are presented as the percentage reduction in the area under the glycemic curve (AUC) relative to the corresponding control group. Group size n = 7. MF—metformin.

To evaluate the hypoglycemic effects of the tested compounds, two oral glucose tolerance tests (OGTT) were conducted within each experiment: the first one was performed two weeks after the start of the experiment with prior administration of the compounds, and the second one was carried out four weeks after the start (after cessation of compound administration). Based on the average data presented as the percentage decrease in blood glucose levels relative to the parameter of the corresponding control group (Figure 5), it is evident that all tested compounds exhibited hypoglycemic activity, which increased by the time of the second OGTT for nearly all compounds (except for **5a**,**h**). Overall, compounds **5f** and **5h** demonstrated the most pronounced hypoglycemic activity in the OGTT. The reference drug metformin showed high activity in the first OGTT but almost completely lost its effect in the second test, which was conducted without prior compound administration (30 min before glucose administration), indicating the absence of a cumulative effect.

#### 2.3.3. ITT

**Figure 6:**
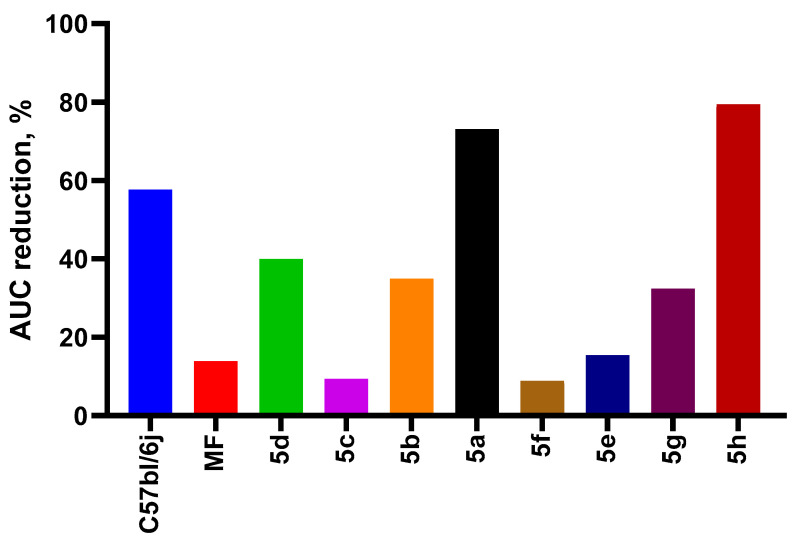
Results of ITT conducted after 4 weeks of compound administration. Data are presented as the percentage reduction in the area under the glycemic curve (AUC) relative to the corresponding control group. Group size n = 7. MF—metformin.

The effect of the tested compounds on insulin sensitivity in experimental animals was assessed using an insulin tolerance test conducted after the administration phase (in the 5th week from the start of the experiment). According to the data obtained by calculating the percentage reduction in the area under the glycemic curve relative to the parameter of the corresponding control group (Figure 6), compounds **5a**,**h** were found to enhance tissue insulin sensitivity to the greatest extent, with their AUC reduction values being comparable to or even exceeding the one of intact mice without obesity and type 2 diabetes (C57Bl/6J). Compounds **5b**,**d**,**g** exhibited a somewhat less pronounced effect, with their AUC values in each experiment (Figure S7) being significantly lower than those of the respective control group. It should also be noted that the reference drug metformin and compounds **5c**,**e**,**f** had minimal effects on tissue insulin sensitivity.

#### 2.3.4. Biochemical Analysis

**Figure 7:**
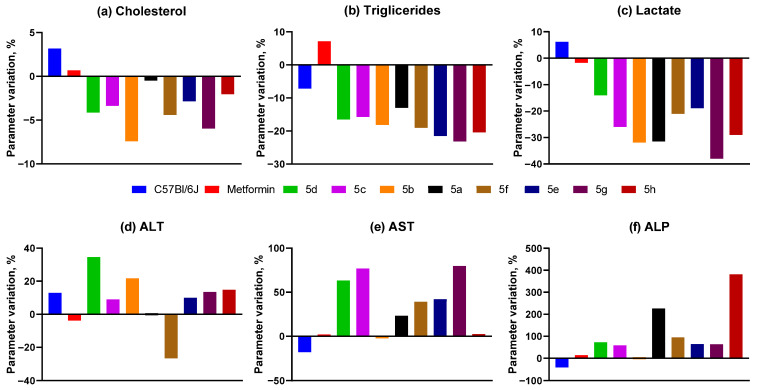
Results of biochemical analysis after the cessation of compound administration. (**a**) Total cholesterol, (**b**) Triglycerides, (**c**) Lactate, (**d**) Alanine aminotransferase (ALT), (**e**) Aspartate aminotransferase (AST), (**f**) Alkaline phosphatase (ALP). Data are presented as the percentage change in blood biochemical parameters relative to the same parameter of the corresponding control group. Group size n = 7.

Biochemical analysis was performed to evaluate the main parameters related to lipid and carbohydrate metabolism (total cholesterol, triglycerides, lactate levels), as well as indicators reflecting liver function (ALT, AST, ALP). The obtained data indicate that all tested compounds (except for compound **5a**) exhibited a hypolipidemic effect, as all of them reduced cholesterol and triglyceride levels (Figure 7a,b). Additionally, administration of all studied compounds led to a statistically significant decrease in blood lactate levels compared to the control group (AY) (Figures S8–S11). Overall, the most pronounced reduction in these parameters was observed in the groups treated with compounds **5b**,**g**. Regarding the liver enzymes, all tested compounds exerted variable and generally mild effects on their activity in the blood. Changes in ALT and AST activity did not exceed a twofold difference relative to the control group. The most significant change in alkaline phosphatase activity was observed in the groups treated with compounds **5a** and **5h**, which increased this parameter by 3- and nearly 5-fold, respectively.

#### 2.3.5. Mass of Animal Organs and Tissues

**Figure 8:**
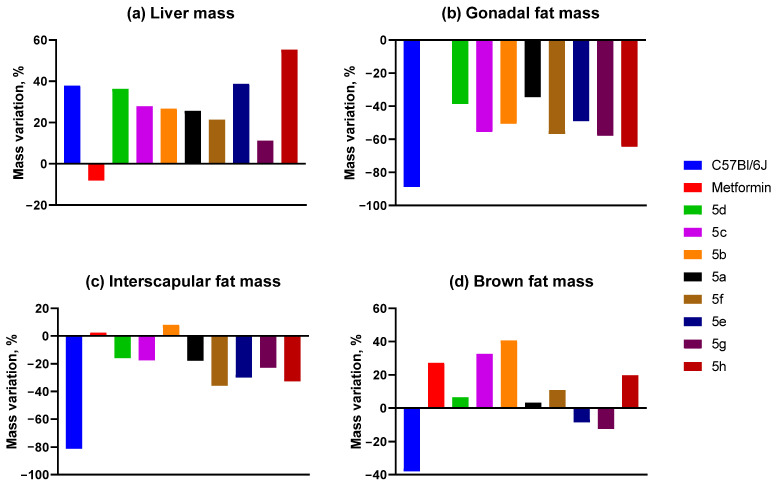
Mass of liver and adipose tissue in animals. (**a**) liver mass, (**b**) gonadal fat mass, (**c**) interscapular fat mass, (**d**) brown adipose tissue mass. Data are presented as the percentage change in mass parameters relative to the corresponding control group. Group size n = 7.

At the end of the experiment, liver mass and the mass of various adipose tissues (gonadal, interscapular white, and brown fat) were assessed in all experimental mice (Figure 8). It was found that liver mass was elevated in all treatment groups compared to the control group (AY). The most pronounced increase in this parameter was observed in animals receiving compounds **5d**,**e**,**h**, whereas the smallest increase was recorded in the group treated with compound **5g**, with no statistically significant difference in liver mass compared to the corresponding control group (Figure S12).

The data on gonadal and interscapular white adipose tissue mass were generally similar across all groups treated with compounds **5a**–**h**. A consistent reduction in these parameters was observed relative to the corresponding control groups, which aligns with the above-mentioned body weight dynamics. The only exception was the group treated with compound **5b**, where a slight increase in interscapular white fat mass was noted.

The effect of the experimental compounds on brown adipose tissue mass was more variable. Some compounds (**5b**,**c**,**f**,**h**), as well as metformin, led to an increase in this parameter, while others (**5e**,**g**) caused a reduction. However, it is important to note that none of the compounds produced statistically significant differences in brown fat mass compared to the control group in any of the experiments (Figures S12–S15).

#### 2.3.6. Histology

**Figure 9:**
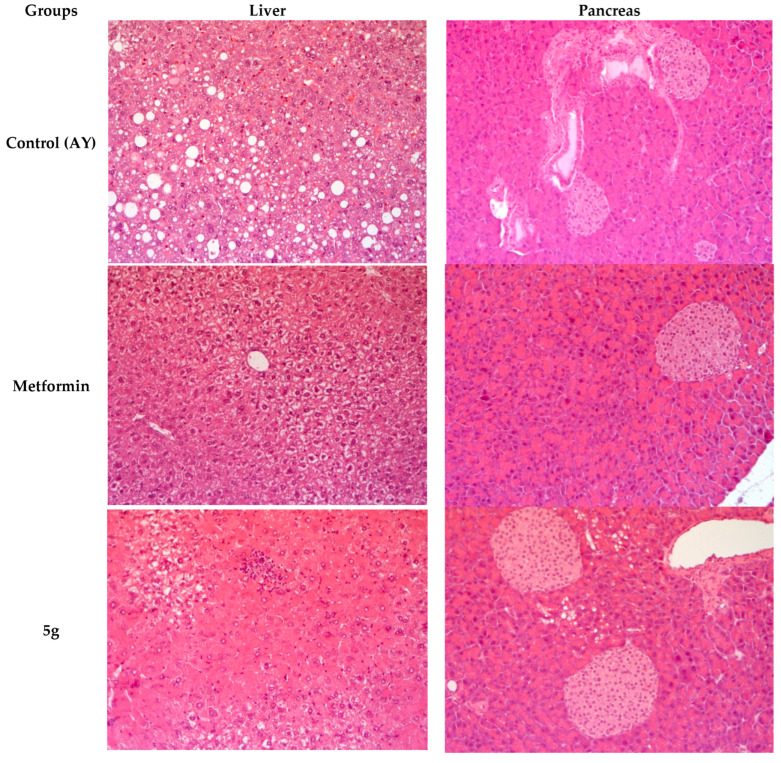

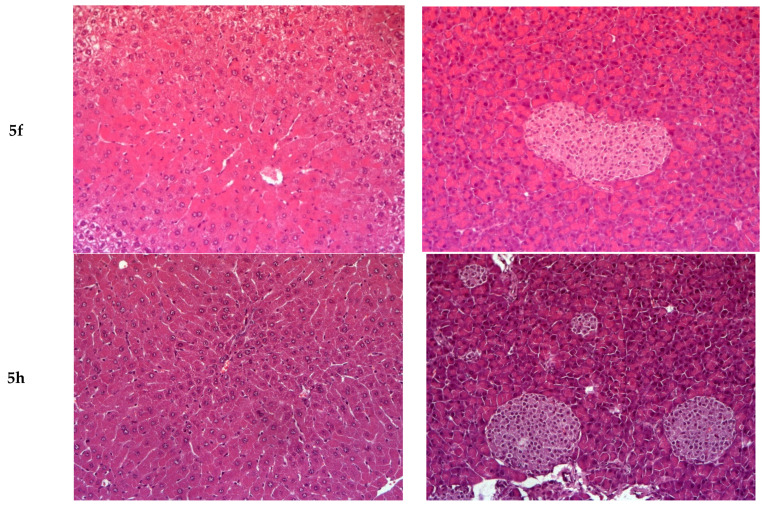
Histological evaluation of liver and pancreas in mice after 4 weeks of the experiment. Hematoxylin and eosin staining. ×200. The presented images are representative for n = 3 repeats of the histological analysis for each organ of each animal.

**Figure 10:**
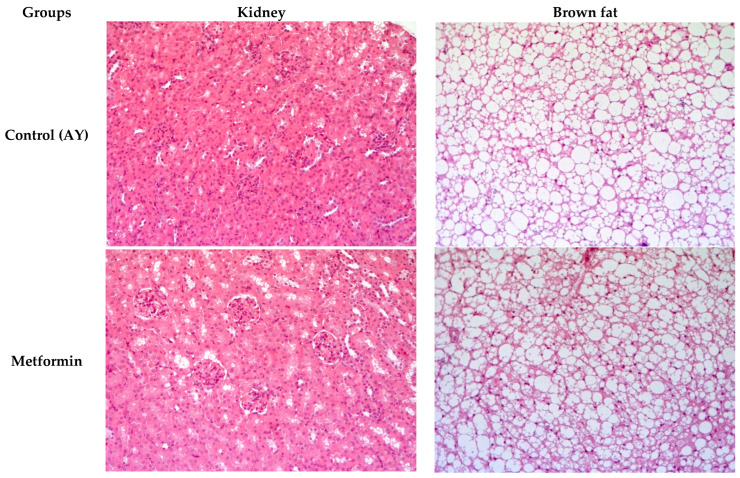

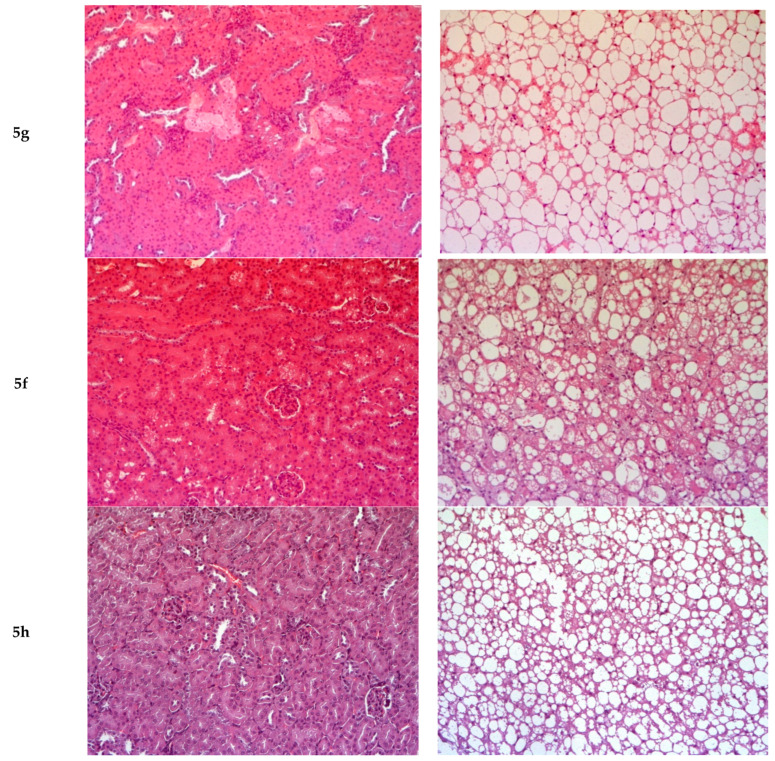
Histological evaluation of the kidney and brown fat in mice after 4 weeks of the experiment. Hematoxylin and eosin staining. ×200. The presented images are representative for n = 3 repeats of the histological analysis for each organ of each animal.

Microscopic assessment of tissue specimens showed that the C57BL/6 A^y^/_a_ control mice developed noticeable pathological alterations in several organs, including the liver, kidneys, and pancreas, along with clear signs of disrupted metabolic processes in adipose tissue. The hepatic samples demonstrated widespread fat accumulation, particularly around periportal areas, often associated with vascular disturbances and localized cell necrosis (Figure 9). In the pancreatic endocrine regions, an expansion of the islet structures was evident. Kidney tissues exhibited focal epithelial cell degeneration, swelling, and interstitial vascular congestion (Figure 10). Cells in brown adipose tissue contained lipid cysts formed by merged fat droplets.

In animals treated with the reference compound metformin, moderate improvements were recorded, such as a reduction in islet hypertrophy within the pancreas and smaller lipid inclusions in brown adipocytes (Figure 9 and Figure 10).

Among all tested molecules, the strongest ameliorative outcomes compared to the AY control group were detected in mice receiving compounds **5b**,**e**,**f**,**h**. Liver morphology in these animals showed almost no fatty degeneration (Figure 9), closely resembling that of intact control mice. The kidneys displayed only scattered mild dystrophic changes, while brown adipose tissue contained noticeably smaller lipid inclusions relative to both the untreated and metformin-treated groups (Figure 10). However, the pancreatic islet enlargement remained comparable to that in untreated control group. Compounds **5c**,**d**,**g** exerted milder beneficial actions, as residual degenerative–necrotic foci and cholestatic features were still visible in the liver (Figure 9). Treatment with compound **5a** resulted in the weakest response: histological profiles of the liver and kidneys were practically indistinguishable from those seen in metformin group. The islet apparatus remained similarly hyperplastic, though slightly less compared to the control group (AY), and no differences in brown adipose tissue morphology were detected.

## 3. Discussion

### 3.1. Chemistry

A set of monoterpenoid esters **5a**–**h** (Scheme 1), containing the *(S)*-2-ethoxy-3-phenylpropanoic acid moiety as the PPAR pharmacophore, was synthesized starting from 4-(2-hydroxyethyl)phenol in five steps. Monoterpene alcohols of various structures were selected for the synthesis: acyclic (geraniol, nerol, citronellol), monocyclic (perillyl alcohol), and bicyclic (myrtenol, nopol). Also, to identify the influence of the structural features of the terpene moiety, the saturated citronellol analog 3,7-dimethyloctanol and the perillyl alcohol aromatic analog—p-cymen-7-ol—were used.

The design of the target compounds was based on avoiding the binding of the monoterpene moiety to the pharmacophore via the amino group, as we believe that protonation of the secondary nitrogen atom was responsible for the complete loss of affinity of the monoterpenoid derivatives we synthesized previously [26] for PPARs, and consequently, the loss of hypoglycemic and lipid-lowering properties.

In the compounds (**5a**–**h**) synthesized in this study, the monoterpene alcohol moiety is linked to the pharmacophore core via an ether bond, without an aminoethanol spacer. An important advantage of the employed approach is its flexibility. It allows for the easy variation in the terpenoid substituent at the penultimate synthesis step, facilitating the targeted preparation of new derivatives and enabling the establishment of structure-activity relationships (SAR) within this compound class.

### 3.2. Biology

In vitro evaluation of PPAR receptor activation revealed that most of the synthesized compounds displayed dual agonistic activity towards PPARα and PPARγ when tested at a concentration of 1 µM. These results were obtained following preliminary cytotoxicity screening using the MTT assay. All substances, except compound **5a**, showed activation of PPARα comparable to that of tesaglitazar, a known dual agonist used as the reference drug [27]. In contrast, PPARγ activation was not significant for compounds **5a**,**b**, while compounds **5c**–**h** demonstrated activation levels similar to rosiglitazone, a selective PPARγ agonist [28]. Among these, compounds **5c**,**d**,**f**–**h** exhibited the strongest combined activity toward both receptor subtypes. With the exception of **5c**, these substances activated PPARα more effectively than PPARγ, a pattern generally considered advantageous because it may lessen the probability of adverse reactions commonly associated with a stronger PPARγ agonism [29].

To further investigate their metabolic effects, the compounds were tested in vivo using the C57BL/6 A^y^/_a_ mouse model. This strain carries a mutation in the agouti gene that suppresses melanocortin receptor function, eventually leading to obesity and hyperinsulinemia—conditions resembling type 2 diabetes mellitus (T2DM) [30]. Therefore, these animals provide a suitable model for assessing both hypolipidemic and hypoglycemic properties of potential antidiabetic agents. Doses for daily administration were determined according to previous experiments with structurally related molecules [15,16].

Experimental results indicated that all glitazar-type derivatives containing a monoterpenoid moiety caused significant body weight reduction throughout the treatment period. The decrease corresponded with reduced masses of gonadal and white interscapular adipose tissue, except in the group receiving compound **5b**. Biochemical analyses confirmed these findings, showing lower plasma levels of cholesterol and triglycerides in animals treated with compounds **5a**–**h** compared to the control ones. Histological observations supported these findings: with the exception of **5a**, all compounds alleviated hepatic steatosis and decreased the size of lipid droplets within brown adipose tissue. Such physiological effects are typical of PPARα agonists, including fibrates and other glitazars [31].

Regarding carbohydrate metabolism, all compounds produced a marked hypoglycemic response that persisted even after treatment cessation. This contrasted with the reference drug metformin, whose effect diminished within 24 h of withdrawal, as shown in the repeated oral glucose tolerance test (OGTT). Metformin served as a comparator due to its well-established role as a first-line T2DM medication that enhances insulin sensitivity and inhibits hepatic gluconeogenesis [32]. The cumulative effect of the tested substances was supported by the fact, that all derivatives except **5a** demonstrated more effective glucose lowering during the second OGTT compared to the first. Because PPARγ activation generally enhances insulin sensitivity [31], insulin tolerance tests (ITT) were conducted to confirm this mechanism. The ITT results revealed that compounds **5a**,**h** were the most effective in reducing insulin resistance. The high PPARγ affinity of **5h** observed in vitro supports this finding. Moreover, the strongest decreases in blood lactate levels were also recorded for compounds with the most pronounced insulin-sensitizing effects. Since elevated lactate concentrations are produced by adipose tissue during obesity and are closely linked with insulin resistance [33], these reductions further confirm the improved tissue responsiveness to insulin.

Additionally, within the scope of this study, several parameters, related to the potential toxicity of the tested compounds, were evaluated. In the biochemical analyses some groups showed moderate increases in hepatic enzyme activities (ALT, AST, and ALP), but these elevations generally did not exceed 100%, suggesting enhanced hepatic metabolism rather than overt toxicity. Literature data indicate that clinically significant hepatotoxicity is usually associated with several-fold rises in transaminase activities [34]. ALP levels were notably higher in groups treated with compounds **5a**,**h**—approximately twofold and fourfold higher than the respective control values. However, histopathological assessment distinguished between these outcomes: compound **5a** induced fatty liver changes and focal hepatocellular necrosis, whereas the hepatic tissue in the **5h** group appeared nearly normal, similar to that of intact control mice (C57BL/6J). This difference implies that ALP elevation in **5a**-treated animals likely reflected hepatotoxicity, while that in **5h**-treated mice was unrelated to tissue injury. A comparable pattern emerged in renal samples. Animals receiving compound **5a** displayed clear signs of dystrophy, edema, and vascular disturbances, whereas kidneys from the **5h** group showed only mild degenerative changes.

Considering all collected data, compound **5h**, which contains a cumin-derived fragment, emerged as the most promising dual PPARα/γ agonist among the tested substances. It activated both receptors efficiently in vitro and demonstrated significant hypoglycemic and hypolipidemic activities in vivo. The improvement in insulin sensitivity corresponded with PPARγ activation, while lipid-lowering effects reflected the PPARα engagement. Furthermore, compound **5h** considerably improved the condition of organs and tissues affected by pathological changes associated with the development of type 2 diabetes mellitus (T2DM).

Compounds **5d**,**g**, containing perillyl alcohol and dimethyl octanol fragments, respectively, produced comparable but slightly weaker results. They exhibited strong hypolipidemic activity (reduction in blood triglyceride level, body weight, and adipose tissue mass); however, their hypoglycemic effect, and especially insulin sensitivity elevation, was comparatively weaker and, according to the histological data, they corrected the T2DM-related morphological alterations to a lesser degree. The remaining derivatives (**5b**,**c**,**e**,**f**) also lowered body weight and blood triglyceride levels, but their hypoglycemic effects likely arose from mechanisms other than insulin sensitization. Finally, compound **5a** showed minimal PPAR activation and correspondingly weak biological activity, confirming that its structure was least suited for dual receptor agonism.

Based on the analysis of pharmacological data of potential PPARα,γ agonists **5a**–**h**, some conclusions can be drawn: when using unsaturated monoterpene alcohols, especially with the presence of cyclic fragments, an increase in hypoglycemic and hypolipidemic activities is observed, where the most active was the derivative **5h**. In the future, various aromatic and heterocyclic fragments can be used as the variable part of the molecule, for example, substituted phenols, which are present in some PPAR agonists. It is worth noting that in the literature to date there are only a few examples of glitazars, that contain monoterpenoid fragments in their structure. A certain advantage of monoterpene derivatives **5a**–**h** with such a structure is that they are quite convenient to assemble, since the attachment of the monoterpene fragment occurs at the last stage, enabling easy modification.

## 4. Materials and Methods

### 4.1. Chemistry

All synthesized compounds were structurally verified by means of nuclear magnetic resonance (NMR) and high-resolution mass spectrometry (HRMS). ^1^H and ^13^C NMR spectra were recorded in CDCl_3_ using a Bruker AV-400 spectrometer (Bruker Corporation, Billerica, MA, USA) operating at 400.13 and 100.61 MHz, respectively. The residual solvent peaks (δH 7.27 and δC 77.1) were employed as internal references. Chemical shifts are reported in parts per million (ppm), and coupling constants (J) are expressed in hertz (Hz). High-resolution mass spectra were obtained on a DFS Thermo Scientific spectrometer (Thermo Fisher Scientific, Waltham, MA, USA) (electron ionization, 70 eV). Purification of products was achieved by column chromatography on Merck silica gel (particle size 63–200 μm). The reaction progress was monitored by thin-layer chromatography (TLC) using Silica gel 60 F_254_ plates. All commercial reagents and solvents were purchased from Sigma-Aldrich, Acros Organics, and Alfa Aesar, and were used as received unless otherwise noted. *(S)*-Ethyl 2-ethoxy-3-(4-hydroxyphenyl)propanoate was prepared following the previously published procedure [25]. Solvents of reagent grade were freshly distilled prior to use [35]. Detailed synthetic protocols and complete spectral data for all target compounds are provided in the Supplementary Materials.

### 4.2. In Vitro Biological Testing

#### 4.2.1. MTT Assay

The cytotoxic potential of the synthesized compounds was evaluated using the 3-(4,5-dimethylthiazol-2-yl)-2,5-diphenyltetrazolium bromide (MTT) assay [36]. Chinese hamster ovary (CHO) cells, obtained from the Institute of Cytology and Genetics (SB RAS, Novosibirsk, Russia), were maintained under standard culture conditions in high-glucose DMEM (Servicebio, Wuhan, China) supplemented with 10% fetal bovine serum (FBS, Sigma-Aldrich, São Paulo, Brazil), 100 μg/mL streptomycin, and 100 U/mL penicillin (Sigma-Aldrich, Saint Louis, MO, USA). Cultures were incubated at 37 °C in a humidified 5% CO_2_ atmosphere (NuAire Inc., Plymouth, MN, USA). Test compounds were dissolved in dimethyl sulfoxide (DMSO; Amresco, Solon, OH, USA) and added to the cell culture at concentrations of 1, 10, and 100 μM, maintaining a final DMSO content of 0.1%. The same DMSO concentration was used in control wells. Cells were seeded into 96-well microplates (TPP, Trasadingen, Switzerland) at a density of 1 × 10^5^ cells/mL in DMEM containing 10% FBS. After 24 h, the medium was replaced with serum-free DMEM containing the test compounds and incubated for another 24 h. Subsequently, an MTT reagent solution (5 mg/mL; PanReac AppliChem, Darmstadt, Germany) was added to each well at a 1:10 ratio and incubated for 4 h at 37 °C. The supernatant was removed, formazan crystals were dissolved in DMSO, and absorbance was measured at 570 nm using a Multiskan Ascent spectrophotometer (Thermo Labsystems, Helsinki, Finland). Cell viability (%) was expressed relative to the control. Data were calculated as mean ± SEM from two independent experiments (each with four replicates).

#### 4.2.2. Dual-Luciferase Reporter Assay to Study the Compounds’ Activity and Specificity

Evaluation of compound activity and receptor specificity was performed as described in [16]. Transgenic CHO-K1 cells co-expressing human hPPARα/hRXRα (CHO-PPARα) or hPPARγ/hRXRα (CHO-PPARγ) were seeded at 2 × 10^4^ cells per well in 96-well opaque plates (Greiner Bio-One, cat. 655098) containing growth medium (DMEM/F12 with 10% FBS, 10 U/mL penicillin, and 10 μg/mL streptomycin). After 24 h, the reporter plasmid PPRE-pGK4.10[Luc2] [16] and the normalization vector pGL4.73[hRluc_SV40] (Promega, Madison, WI, USA) were introduced using Lipofectamine 3000 (Thermo Fisher Scientific, Waltham, MA, USA). Twenty-four hours post-transfection, the culture medium was replaced with HBSS (Thermo Fisher Scientific) supplemented with reference and experimental compounds (1 μM each, in 0.1% DMSO). The PPARγ agonist rosiglitazone and the dual PPARα/γ agonist tesaglitazar (both from Sigma-Aldrich, Darmstadt, Germany) were used as positive controls, while 0.1% DMSO served as the negative control. After 24 h of exposure, cells were lysed, and luciferase activity was quantified using the Dual-Luciferase Reporter Assay System (Promega, Madison, WI, USA) on a ClarioStar Plus (BMG LABTECH, Ortenberg, Germany) plate reader equipped for dual-injection detection, following the manufacturer’s protocol.

### 4.3. In Vivo Experiments

#### 4.3.1. Animals

Male C57BL/6 A^y^/_a_ (AY) mice weighing 28–32 g and male C57BL/6J mice weighing 20–25 g were utilized in this study. Animals were obtained from the specific-pathogen-free (SPF) vivarium of the Institute of Cytology and Genetics (SB RAS). They were housed under controlled environmental conditions (temperature, humidity, and 12 h light/dark cycle) with unrestricted access to food and water. All experimental procedures complied with the regulatory standards of the Russian Federation (Decree No. 199n, Ministry of Health, 1 April 2016) and conformed to Directive 2010/63/EU of the European Parliament and Council (22 September 2010) regarding the protection of animals used for scientific purposes. The study protocol was reviewed and approved by the Ethics Committee of the N.N. Vorozhtsov Institute of Organic Chemistry, SB RAS (Protocol No. P-14-2024-11-01).

#### 4.3.2. The OGTT

Before testing, animals were deprived of food for 12 h. All groups received an oral glucose dose of 2.5 g/kg, administered 30 min before blood glucose measurement. The first OGTT was carried out in AY mice after 14 days of compound administration. Each test substance was given orally (by gavage) 30 min before the glucose load. A second OGTT was performed on day 28, two days after the final compound administration. Blood samples were collected from the tail vein at 0 (pre-dose), 30-, 60-, 90-, and 120-min post-glucose administration. Blood glucose concentrations were measured with a ONE TOUCH Select glucometer (LIFESCAN Inc., Milpitas, CA, USA). The area under the glycemic curve (AUC) was determined using Tai’s method [37].

#### 4.3.3. The ITT

The ITT was conducted according to the same experimental design used for AY mice. After a 4 h fasting period, insulin (soluble human insulin; Medsynthesis Plant, Novouralsk, Russia) was administered intraperitoneally at a dose of 5 U/kg. Blood samples were collected from the tail vein prior to injection (0 min) and at 15, 30-, 45-, 60-, and 90-min post-injection. Blood glucose levels were measured using a ONE TOUCH Select glucometer (LIFESCAN Inc., Milpitas, CA, USA).

#### 4.3.4. The AY Mice Experiment Design

**Figure 11:**
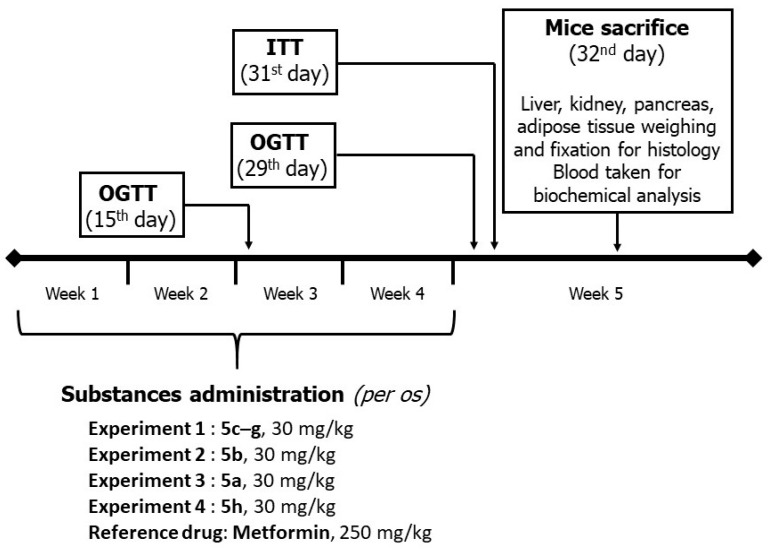
The in vivo experimental design.

Four independent experiments were performed to assess the biological effects of the test compounds. Obesity was induced in C57BL/6 Ay/a mice by feeding a high-calorie diet consisting of standard chow supplemented with lard and cookies ad libitum for 30 days. Animals that reached a body weight of at least 35 g were selected for further experimentation. Selected mice were randomly divided into groups (n = 7 per group) for each study as follows: Experiment 1—(1) Control (vehicle (water + 2 drops of Tween 80)), (2) MF 250 mg/kg, (3–7) **5c**–**g**, 30 mg/kg; Experiment 2—(1) C57BL/6 mice, (n = 7), (2) Control (vehicle (water + 2 drops of Tween 80)), (3) MF 250 mg/kg, (4) **5b**, 30 mg/kg; Experiment 3—(1) Control (vehicle (water + 2 drops of Tween 80)), (2) MF 250 mg/kg, (3) **5a**, 30 mg/kg; Experiment 4—(1) C57BL/6 mice, (n = 7), (2) Control (vehicle (water + 2 drops of Tween 80)), (3) MF 250 mg/kg, (4) **5h**, 30 mg/kg. Throughout the study, all animals were maintained on the same diet used during the weight-gain phase. The dose of metformin was selected according to literature data [38], while doses of the potential glitazar analogs were chosen based on our previous studies on structurally similar compounds [14,16]. Test compounds were administered once daily by oral gavage (Figure 11). OGTTs were conducted on days 14 and 28. On day 31, animals were euthanized by decapitation, and blood samples were collected for biochemical analysis. For histological examination, the liver, kidneys, gonadal fat, interscapular white and brown adipose tissues, and pancreas were harvested. Food intake and body weight were recorded once at the start of every week.

#### 4.3.5. Biochemical Assays

Blood serum was isolated by centrifugation at 1640× *g* for 15 min. Biochemical parameters were analyzed using standard diagnostic kits (Vector-Best, Novosibirsk, Russia) and a Multiskan Ascent microplate photometer (Thermo Labsystems, Helsinki, Finland). The following serum indicators were determined: total cholesterol, triglycerides, and lactate levels, along with the enzymatic activities of alkaline phosphatase (ASP), alanine aminotransferase (ALT), and aspartate aminotransferase (AST).

#### 4.3.6. Histological Examination

Excised tissues (liver, kidneys, interscapular white and brown adipose tissue, and pancreas) were fixed in 10% neutral buffered formalin for 7 days, followed by standard dehydration through ascending ethanol solutions and xylene. The tissue samples were embedded in paraffin using an AP 280 workstation with Histoplast (Thermo Fisher Scientific, Waltham, MA, USA; melting point = 58 °C). Sections with a thickness of 4.5 μm were prepared using an NM 335E rotary microtome equipped with disposable blades. The sections were stained with hematoxylin and eosin, and examined under a Carl Zeiss Axioscope 40 light microscope (Carl Zeiss, Goettingen, Germany) at magnifications ranging from ×100 to ×400.

#### 4.3.7. Statistical Analysis

Statistical analysis of the in vitro PPARα and PPARγ receptor agonist activities was carried out using the Wilcoxon test with Bonferroni correction for multiple comparisons. Data from in vivo experiments were analyzed using the Mann–Whitney U test and one-way ANOVA followed by Dunnett’s multiple comparisons test. Results are expressed as mean ± SEM. Differences were considered to have statistical significance at *p* < 0.05.

## 5. Conclusions

For the purpose of searching for new effective and safe dual PPARα/γ agonists we synthesized 8 compounds, containing monoterpenoids as the variable moiety which are directly conjugated with the fixed pharmacophoric core. In vitro evaluation of the compounds’ activity towards PPARα/γ showed, that almost all of them except for **5a** and **5b** could be classified as dual PPARα/γ agonists. The in vivo study of the compounds’ antidiabetic action in C57BL/6 A^y^/_a_ mice showed, that according to the results of both OGTTs, all substances showed some level of hypoglycemic activity, the most active of them being **5d**, **5f** and **5h**. Yet, among them, as was shown in the ITT, only in compounds’ **5h** group this effect was primarily due to elevation of insulin sensitivity, a characteristic feature of PPARγ agonists. As far as lipid metabolism is concerned, almost every tested compound (except for **5a**) showed pronounced hypolipidemic activity manifested by adipose tissue and, therefore, body mass reduction as well as blood cholesterol and triglyceride levels decrease compared to control C57BL/6 A^y^/_a_ animals. However, the histological investigation showed that the pathological alterations specific for T2DM in the liver, kidney and brown adipose tissue were corrected the most only in animals treated with **5b**, **e**, **f** and **h**.

Overall, it can be stated that monoterpenoids, as part of the variable moiety of dual PPARα/γ agonists, may have a significant positive impact on the efficiency and safety on this class of drugs. In the present study, all things considered, **5h** was the most favorable of all the tested substances, exhibiting pronounced hypoglycemic and hypolipidemic activities, that were directly connected to its high level of PPARα and PPARγ activation, combined with a general beneficial effect on all the parameters, that could be potentially connected to toxicity.

## Figures and Tables

**Table 1:** The results of MTT assay.

		Cell Viability, %	
Group	1 µM	10 µM	100 µM
**5c**	112.6 ± 7.2	6.0 ± 1.3	2.9 ± 0.8
**5b**	110.8 ± 6.4	2.0 ± 0.4	2.7 ± 0.6
**5f**	106.0 ± 14.1	3.5 ± 0.6	2.9 ± 0.6
**5g**	113.1 ± 19.5	115.2 ± 20.4	2.5 ± 0.8
**5h**	115.8 ± 4.8	3.8 ± 1.3	2.8 ± 0.5

## Data Availability

The original contributions presented in this study are included in the article. Further inquiries can be directed to the corresponding author.

## Supplementary Materials

The following supporting information can be downloaded at: https://www.mdpi.com/article/10.3390/molecules30244775/s1, Figure S1: Dynamics of changes in body weight in mice during every experiment, Figure S2: Glycemic curves of the performed OGTTs after two weeks of each experiment, Figure S3: Areas under the glycemic curves (AUC) of the performed OGTTs after two weeks of each experiment, Figure S4: Glycemic curves of the performed OGTTs after four weeks of each experiment, Figure S5: Areas under the glycemic curves (AUC) of the performed OGTTs after four weeks of each experiment, Figure S6: Glycemic curves of the performed ITTs, Figure S7: Areas under the glycemic curves (AUC) of the performed ITTs, Figure S8: Biochemical blood parameters after the end of compounds **5c**–**g** administration, Figure S9: Biochemical blood parameters after the end of compound **5b** administration, Figure S10: Biochemical blood parameters after the end of compounds **5a** administration, Figure S11: Biochemical blood parameters after the end of compounds **5h** administration, Figure S12: Liver and adipose tissue weight of animals (Compounds **5a**–**g**), Figure S13: Liver and adipose tissue weight of animals (Compound **5b**), Figure S14: Liver and adipose tissue weight of animals (Compound **5a**), Figure S15: Liver and adipose tissue weight of animals (Compound **5h**), Figure S16: ^1^H-NMR spectrum of Ethyl *(2S)*-3-{4-[2-(4-{[(2E)-3,7-dimethylocta-2,6-dien-1-yl]oxy}phenyl)ethoxy]phenyl}-2-ethoxypropanoate (**4a**), Figure S17: ^13^C-NMR spectrum of Ethyl *(2S)*-3-{4-[2-(4-{[(2E)-3,7-dimethylocta-2,6-dien-1-yl]oxy}phenyl)ethoxy]phenyl}-2-ethoxypropanoate (**4a**), Figure S18: ^1^H-NMR spectrum of Ethyl *(2S)*-3-{4-[2-(4-{[(1R,5S)-6,6-dimethylbicyclo [3.1.1]hept-2-en-2-yl]methoxy}phenyl)ethoxy]phenyl}-2-ethoxypropanoate (**4b**), Figure S19: ^13^C-NMR spectrum of Ethyl *(2S)*-3-{4-[2-(4-{[(1R,5S)-6,6-dimethylbicyclo [3.1.1]hept-2-en-2-yl]methoxy}phenyl)ethoxy]phenyl}-2-ethoxypropanoate (**4b**), Figure S20: ^1^H-NMR spectrum of Ethyl *(2S)*-3-{4-[2-(4-{2-[(1R,5S)-6,6-dimethylbicyclo [3.1.1]hept-2-en-2-yl]ethoxy}phenyl)ethoxy]phenyl}-2-ethoxypropanoate (**4c**), Figure S21: ^13^C-NMR spectrum of *(2S)*-3-{4-[2-(4-{2-[(1R,5S)-6,6-dimethylbicyclo [3.1.1]hept-2-en-2-yl]ethoxy}phenyl)ethoxy]phenyl}-2-ethoxypropanoate (**4c**), Figure S22: ^1^H-NMR spectrum of Ethyl *(2S)*-3-[4-(2-{4-[(3,7-dimethyloctyl)oxy]phenyl}ethoxy)phenyl]-2-ethoxypropanoate (**4d**), Figure S23: ^13^C-NMR spectrum of Ethyl *(2S)*-3-[4-(2-{4-[(3,7-dimethyloctyl)oxy]phenyl}ethoxy)phenyl]-2-ethoxypropanoate (**4d**), Figure S24: ^1^H-NMR spectrum of Ethyl *(2S)*-3-[4-(2-{4-[(3,7-dimethyloct-6-en-1-yl)oxy]phenyl}ethoxy)phenyl]-2-ethoxypropanoate (**4e**), Figure S25: ^13^C-NMR spectrum of Ethyl *(2S)*-3-[4-(2-{4-[(3,7-dimethyloct-6-en-1-yl)oxy]phenyl}ethoxy)phenyl]-2-ethoxypropanoate (**4e**), Figure S26: ^1^H-NMR spectrum of Ethyl *(2S)*-3-{4-[2-(4-{[(2Z)-3,7-dimethylocta-2,6-dien-1-yl]oxy}phenyl)ethoxy]phenyl}-2-ethoxypropanoate (**4f**), Figure S27: ^13^C-NMR spectrum of Ethyl *(2S)*-3-{4-[2-(4-{[(2Z)-3,7-dimethylocta-2,6-dien-1-yl]oxy}phenyl)ethoxy]phenyl}-2-ethoxypropanoate (**4f**), Figure S28: ^1^H-NMR spectrum of Ethyl *(2S)*-2-ethoxy-3-{4-[2-(4-{[4-(prop-1-en-2-yl)cyclohex-1-en-1-yl]methoxy}phenyl)ethoxy]phenyl}propanoate (**4g**), Figure S29: ^13^C-NMR spectrum of Ethyl *(2S)*-2-ethoxy-3-{4-[2-(4-{[4-(prop-1-en-2-yl)cyclohex-1-en-1-yl]methoxy}phenyl)ethoxy]phenyl}propanoate (**4g**), Figure S30: ^1^H-NMR spectrum of Ethyl *(2S)*-2-ethoxy-3-{4-[2-(4-{[4-(propan-2-yl)phenyl]methoxy}phenyl)ethoxy]-phenyl}propanoate (**4h**), Figure S31: ^1^H-NMR spectrum of *(2S)*-3-{4-[2-(4-{[(2E)-3,7-dimethylocta-2,6-dien-1-yl]oxy}phenyl)ethoxy]phenyl}-2-ethoxypropanoic acid (**5a**), Figure S32: ^13^C-NMR spectrum of *(2S)*-3-{4-[2-(4-{[(2E)-3,7-dimethylocta-2,6-dien-1-yl]oxy}phenyl)ethoxy]phenyl}-2-ethoxypropanoic acid (**5a**), Figure S33: ^1^H-NMR spectrum of *(2S)*-3-{4-[2-(4-{[(1R,5S)-6,6-dimethylbicyclo [3.1.1]hept-2-en-2-yl]methoxy}phenyl)ethoxy]phenyl}-2-ethoxypropanoic acid (**5b**), Figure S34: ^13^C-NMR spectrum of *(2S)*-3-{4-[2-(4-{[(1R,5S)-6,6-dimethylbicyclo [3.1.1]hept-2-en-2-yl]methoxy}phenyl)ethoxy]phenyl}-2-ethoxypropanoic acid (**5b**), Figure S35: ^1^H-NMR spectrum of *(2S)*-3-{4-[2-(4-{2-[(1R,5S)-6,6-dimethylbicyclo [3.1.1]hept-2-en-2-yl]ethoxy}phenyl)ethoxy]phenyl}-2-ethoxypropanoic acid (**5c**), Figure S36: ^1^H-NMR spectrum of *(2S)*-3-[4-(2-{4-[(3,7-dimethyloctyl)oxy]phenyl}ethoxy)phenyl]-2-ethoxypropanoic acid (**5d**), Figure S37: ^13^C-NMR spectrum of *(2S)*-3-[4-(2-{4-[(3,7-dimethyloctyl)oxy]phenyl}ethoxy)phenyl]-2-ethoxypropanoic acid (**5d**), Figure S38: ^1^H-NMR spectrum of *(2S)*-3-[4-(2-{4-[(3,7-dimethyloct-6-en-1-yl)oxy]phenyl}ethoxy)phenyl]-2-ethoxypropanoic acid (**5e**), Figure S39: ^1^H-NMR spectrum of *(2S)*-3-[4-(2-{4-[(3,7-dimethyloct-6-en-1-yl)oxy]phenyl}ethoxy)phenyl]-2-ethoxypropanoic acid (**5e**), Figure S40: ^1^H-NMR spectrum of *(2S)*-3-{4-[2-(4-{[(2Z)-3,7-dimethylocta-2,6-dien-1-yl]oxy}phenyl)ethoxy]phenyl}-2-ethoxypropanoic acid (**5f**), Figure S41: ^13^C-NMR spectrum of *(2S)*-3-{4-[2-(4-{[(2Z)-3,7-dimethylocta-2,6-dien-1-yl]oxy}phenyl)ethoxy]phenyl}-2-ethoxypropanoic acid (**5f**), Figure S42: ^1^H-NMR spectrum of *(2S)*-2-ethoxy-3-{4-[2-(4-{[4-(prop-1-en-2-yl)cyclohex-1-en-1-yl]methoxy}phenyl)ethoxy]phenyl}propanoic acid (**5g**), Figure S43: ^1^H-NMR spectrum of *(2S)*-2-ethoxy-3-{4-[2-(4-{[4-(prop-1-en-2-yl)cyclohex-1-en-1-yl]methoxy}phenyl)ethoxy]phenyl}propanoic acid (**5g**), Figure S44: ^1^H-NMR spectrum of *(2S)*-2-ethoxy-3-{4-[2-(4-{[4-(propan-2-yl)phenyl]methoxy}phenyl)ethoxy]-phenyl}propanoic acid (**5h**), Figure S45: HPLC spectrum of *(2S)*-3-{4-[2-(4-{[(2E)-3,7-dimethylocta-2,6-dien-1-yl]oxy}phenyl)ethoxy]phenyl}-2-ethoxypropanoic acid (**5a**), Figure S46: HPLC spectrum of *(2S)*-3-{4-[2-(4-{[(1R,5S)-6,6-dimethylbicyclo [3.1.1]hept-2-en-2-yl]methoxy}phenyl)ethoxy]phenyl}-2-ethoxypropanoic acid (**5b**), Figure S47: HPLC spectrum of *(2S)*-3-{4-[2-(4-{2-[(1R,5S)-6,6-dimethylbicyclo [3.1.1]hept-2-en-2-yl]ethoxy}phenyl)ethoxy]phenyl}-2-ethoxypropanoic acid (**5c**), Figure S48: HPLC spectrum of *(2S)*-3-[4-(2-{4-[(3,7-dimethyloctyl)oxy]phenyl}ethoxy)phenyl]-2-ethoxypropanoic acid (**5d**), Figure S49: HPLC spectrum of *(2S)*-3-[4-(2-{4-[(3,7-dimethyloct-6-en-1-yl)oxy]phenyl}ethoxy)phenyl]-2-ethoxypropanoic acid (**5e**), Figure S50: HPLC spectrum of *(2S)*-3-{4-[2-(4-{[(2Z)-3,7-dimethylocta-2,6-dien-1-yl]oxy}phenyl)ethoxy]phenyl}-2-ethoxypropanoic acid (**5f**), Figure S51: HPLC spectrum of *(2S)*-2-ethoxy-3-{4-[2-(4-{[4-(prop-1-en-2-yl)cyclohex-1-en-1-yl]methoxy}phenyl)ethoxy]phenyl}propanoic acid (**5g**), Figure S52: HPLC spectrum of *(2S)*-2-ethoxy-3-{4-[2-(4-{[4-(propan-2-yl)phenyl]methoxy}phenyl)ethoxy]-phenyl}propanoic acid (**5h**).

## Abbreviations

The following abbreviations are used in this manuscript:

LDL	Low-Density Lipoprotein
T2DM	Type 2 Diabetes Mellitus
DMSO	Dimethyl Sulfoxide
PPAR	Peroxisome Proliferator-Activated Receptors
DIAD	Diisopropyl Azodicarboxylate
THF	Tetrahydrofuran
HPLC	High-Performance Liquid Chromatography
NMR	Nuclear magnetic resonance
OGTT	Oral Glucose Tolerance Test
AUC	Area Under the Curve
ITT	Insulin Tolerance Test
ALT	Alanine Aminotransferase
AST	Aspartate Aminotransferase
ALP	Alkaline Phosphatase
